# Authentication of Indian Honey Based on Carbon Stable Isotope Ratio Analysis—Verification of Indian Regulatory Criteria

**DOI:** 10.3390/foods14081289

**Published:** 2025-04-08

**Authors:** Ajit Dua, Sanjivan Bahman, Simon Kelly, Shainandni Dogra, Kirti Sharma

**Affiliations:** 1 Punjab Biotechnology Incubator, Knowledge City, Sector 81, Sahibzada Ajit Singh Nagar 140306, Punjab, India; 2 International Atomic Energy Agency, Vienna International Centre, Wagramer Strasse 5, P.O. Box 100, 1400 Vienna, Austria

**Keywords:** honey, carbon stable isotope (^13^C/^12^C), botanical source and geographical regions, honey authenticity, adulteration, EA/LC-IRMS

## Abstract

The present study was undertaken for the first time in India to generate a database of isotopic signatures of authentic Indian honey to verify the regulatory criteria laid down by the Food Safety and Standards Authority of India (FSSAI). In this study, ninety-eight (98) authentic honey samples from nineteen (19) different botanical sources were collected from five (05) geographical regions of India and analyzed to generate a database of stable carbon isotope ratios (^13^C/^12^C) by Elemental Analyzer/Liquid Chromatography–Isotopic Ratio Mass Spectrometry (EA/LC-IRMS). The samples were analyzed for the parameters *δ*^13^C_Honey_(*δ*^13^C_H_), *δ*^13^C_Protein_(*δ*^13^C_P_), *δ*^13^C individual sugars, ∆*δ*^13^C_Protein-Honey_(*δ*^13^C_P-H_), C4 sugar, ∆*δ*^13^C_Fructose-Glucose_(*δ*^13^C_Fru-Glu_), ∆*δ*^13^C_max_, and foreign oligosaccharides (FOs), as per the official methods of analysis of the Association of Official Analytical Chemists (AOAC 998.12) and the FSSAI. The results were evaluated against the published literature and Indian regulatory criteria for authentic honey. The *δ*^13^C value for honey (*δ*^13^C_H_) ranged from −22.07 to −29.02‰. It was found that 94% of Indian honey samples met the criteria for Δ*δ*^13^C_P-H_ (≥−1.0‰), Δ*δ*^13^C_Fru-Glu_ (±1.0‰), and C4 sugar content (7% maximum), with negative C4 sugar values treated as 0% as prescribed by the AOAC method. Further, 86% of samples met the FO criteria (maximum 0.7% peak area). Thus, the data of this study provide scientific backing for these four (04) parameters as per the FSSAI regulation. However, the non-compliance of a high number (47%) of authentic honey samples for Δ*δ*^13^C_max_ (±2.1‰) compels further systematic investigation with a special focus on bee feeding practices. Further, in the present study, it was found that honey samples with a Δ*δ*^13^C_P-H_ greater than +1‰ and a C4 sugar content more negative than −7% also did not comply with the Δ*δ*^13^C_max_ criteria. Hence, Δ*δ*^13^C_P-H_ values (>+1‰ equivalent to C4 sugar < −7%) could be an indicator of C3 adulteration to some extent.

## 1. Introduction

Honey, a natural sweet and viscous substance produced by honeybees from flower nectar, or honeydew secreted by insects, mainly consists of 33–43% fructose, 25–35% glucose, and 2% sucrose with small amounts of protein, pollen, acids, trace elements, enzymes, vitamins, and flavonoids [1]. Due to its excellent flavor, odor, nutritional value, and healing properties, honey consumption has significantly increased in recent years which has elevated demand worldwide [2]. The popularity of honey has brought challenges such as adulteration and mislabeling, compromising its quality and authenticity at the global level, which has affected consumer perception, trust, brand reputation, and market stability [3,4,5,6]. The major concern remains direct adulteration with inexpensive sweeteners (e.g., invert sugar syrups, corn syrups, high fructose and maltose syrups, rice syrup, beetroot syrup, jaggery syrup, etc.) which mimic the natural sugars in honey and are difficult to detect with traditional chemical methods of analysis. Although these kinds of economically motivated adulterations are not necessarily injurious to health, they can negatively impact market growth by damaging consumers’ confidence [3,4,5,6].

Several analytical techniques, such as HPLC [7], GCMS [8], Mid-Infrared (MIR) spectroscopy [9], and HPAEC-PAD anion exchange chromatography with pulsed amperometric detection [10], NMR [8,9], HPLC-RI [11,12], NIR, Raman PLS-LDA, etc. [13], have been reported for the detection of honey adulteration. With the advancement of analytical methods, the kind of adulteration has also evolved during the last two (02) decades. Recent investigations in the European Union by the Joint Research Center reported that out of the 320 honey samples taken at EU borders, 46% were suspected of being non-compliant with EU Honey Directive 2001/110/EC with respect to authenticity based on EA/LC-IRMS, HPAEC-PAD, LC-HRMS, and ^1^H-NMR spectroscopy methods [14].

In the early 1980s, Stable Carbon Isotope Ratio Analysis (SCIRA) using Elemental Analyzer–Isotopic Ratio Mass Spectrometry (EA-IRMS) [15,16] proved to be a milestone in the detection of the more sophisticated adulteration of honey with high-fructose corn syrup (HFCS). The method is based on the principle that plants that utilize the Calvin–Benson photosynthetic pathway (known as C3 plants), which generally serve as the source of nectar for honey bees, have *δ*^13^C_H_ values ranging from −22 to −32‰, whereas plants using the Hatch–Slack photosynthetic pathway (known as C4 plants, like maize and sugarcane) have *δ*^13^C_H_ values ranging from −8 to −16‰ [15,17]. Thus, the *δ*^13^Cvalue of honey is the basis for the detection of adulteration with C4 plant sugars. As per White et al. [18], the *δ*^13^C value for pure honey should be more negative than −23.5‰, whereas Elflein and Raezke in 2008 [19] reported *δ*^13^C values of pure honey ranging from −23.0‰ to −27.3‰.

The SCIRA method was further improved by using the extracted protein fraction of honey as an internal isotopic standard which lowered the detection limit of C4 sugar from 20 to 7% *w*/*w* [20,21,22]. The AOAC official method 998.12 [20] has been widely adopted by some countries for assessing the authenticity of honey. However, it has limitations in detecting C3 plants-based sugar syrups, e.g., rice and beet sugar syrups, which have isotopic signatures similar to honey because they utilize the C3 photosynthetic pathway. Similarly, certain honeys containing lower amounts of the protein fraction such as Acacia, and lavender honey with a high content of yeast or remnants of bee feedings and a detection limit of C4 sugar up to 7% [19], are the other limiting factors of this technique.

The limitations of detecting C3 sugar adulteration in honey were addressed by Cabanero et al., in 2006 [3], through the method based on Liquid Chromatography coupled to Isotope Ratio Mass Spectrometry, EA/LC-IRMS, which permitted more sensitive detection of C4 and C3 plant-based syrups at 1% and 10% levels of detection, respectively.

Further, Elflein and Raezke in 2008 [19] improved the method of detection of honey adulteration with C3/C4 inverted sugar syrups by using EA/LC-IRMS. The study was conducted on 451 honey samples collected globally, including a few samples from India, and proposed the following criteria: Δ*δ*^13^C_max_ (‰) ± 2.1 (maximum difference between all measured *δ*^13^C values); Δ*δ*^13^C_Fru-Glu_ (‰) ± 1.0; Δ*δ*^13^C_P-H_ (‰) ≥ –1 for authentic honey. The newly developed EA/LC-IRMS method and the purity criteria defined represented a significant improvement compared to existing methods.

The EA-IRMS-based criteria were included in honey regulation by Turkey in 2012 [23], and the EA/LC-IRMS-based criteria were included in Indian regulation in 2018 [24,25], as well as being adopted by the True source certified^®^ Standard in 2024 [26] by trading bodies. Recently, in 2024, an LC-IRMS-based method was also published by the European Committee for Standardization (CEN) and adopted by the British Standards Institution for honey authenticity testing [27].

India, being a country of rich biodiversity with more than 500 plant species, plays a significant role in providing a diverse range of honey and supports the global honey industry, as the seventh-largest producer and third-largest exporter. The Government of India rolled out the National Bee Keeping and Honey mission (NBHM) in 2020 to give a push to the “Sweet Revolution” which was launched to double farmers’ incomes.

The Food Safety and Standards Authority of India (FSSAI) amended the regulatory criteria for honey in 2018, and included isotopic signature based tests for ensuring honey authenticity [25]: C4 sugar, percent by mass, Max. 7.0, Δ*δ*^13^C_P-H_ (‰) ≥ −1.0, Δ*δ*^13^C_Fru-Glu_ (‰) ± 1.0, Δ*δ*^13^C_max_ (‰) ± 2.1, foreign oligosaccharides (Max. Percent Peak Area) 0.7%. The present study has been undertaken for the first time in India to generate a database of isotopic signatures of authentic Indian honey of different botanical and geographical origins and to verify the criteria based on the database generated.

## 2. Materials and Methods

### 2.1. Honey Samples

**Figure 1:**
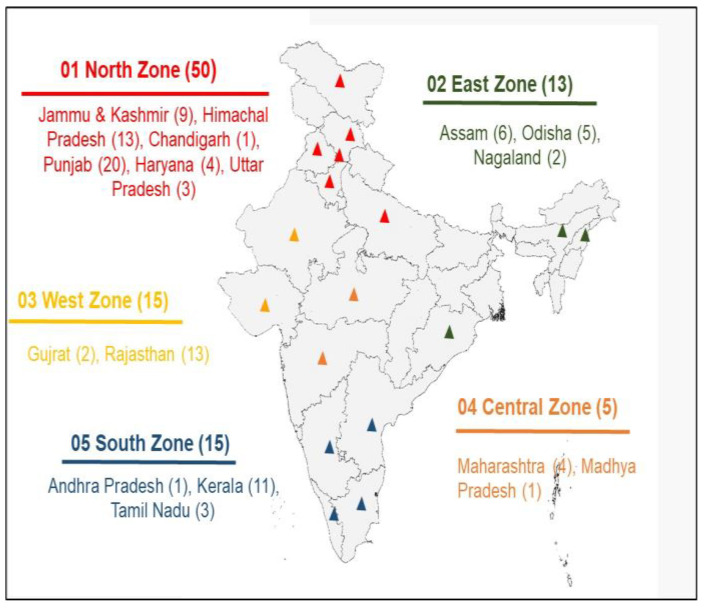
Geographical region- and state-wise details of honey samples.

The centers (26 in number) of the All-India Coordinated Research Project on Honey Bees and Pollinators (AICRP, HB&P), under the auspices of the Indian Council of Agricultural Research (ICAR), all over the country, were identified for the collection of honey samples. In addition, beekeepers registered with the National Bee Board (NBB) were also identified for sample collection. A total of 98 honey samples were collected from 17 states (provinces) of India falling under five (05) geographical regions, viz., east (*n* = 13), north (*n* = 50), west (*n* = 15), south (*n* = 15), and central (*n* = 5) (Figure 1). The collected samples comprised 42% multifloral and 58% monofloral honey samples of 18 different floral sources as detailed in Table 1.

A standard operating procedure was followed for sample collection. All of the required data like the apiary location, floral source, feeding practices, period of harvest, etc., were recorded in field log sheets to ensure traceability to their geographical and botanical origin. All honey samples were collected in glass/food-grade plastic containers and kept at room temperature (25 °C) until analysis.

### 2.2. Reference Materials and Reagents

Reference materials with known values of *δ*^13^C were used during this study. Beet sugar IA-R005^13^C (*δ*^13^C value: −26.03‰) and cane sugar IA-R005^13^C (*δ*^13^C value: −11.64‰) sourced from iso-analytical analysis and casein (*δ*^13^C value –26.98‰) from elemental microanalysis were used for the linear calibration curve in EA-IRMS. For LC-IRMS, BCR-657 glucose (*δ*^13^C value: −10.76‰) procured from the European Commission, Joint Research Center, and other sugar standards of glucose, fructose, sucrose, and raffinose (purity ≥ 99%) from Sigma Aldrich, and calibrated by EA-IRMS, were used for linear delta-scale calibration curve construction. High-purity (>99%) CO_2_ (*δ*^13^C = −26.27‰) was used as a reference gas. The chemicals sodium peroxodisulfate and sodium tungstate dehydrate (Merck, puriss. p.a.  ≥98%), sulfuric acid analytical grade (SD-fine), and ultrapure water (Milli-Q Millipore system < 18.2 MΩ) were used during this study.

### 2.3. Instrumentation and Measurement

The *δ*^13^C values of honey, protein, and individual sugars (disaccharides, fructose, glucose, sucrose, and trisaccharides) were determined by an Isotope Ratio Mass Spectrometer (IRMS) coupled with two CO_2_ preparation devices, one with an Elemental Analyzer (EA-IRMS) and the second with High-Performance Liquid Chromatography (LC-IRMS) via an Isoprimeliquiface.

#### 2.3.1. EA-IRMS

The samples of honey were analyzed for the *δ*^13^C of bulk honey (*δ*^13^C_H_) and its protein fraction (*δ*^13^C_P_), Δ*δ*^13^C_P-H_, and C4 sugar as per the AOAC 998.12 method [20]. The analysis of the *δ*^13^C of bulk honey and its protein fraction was carried out using an IRMS (Make: Isoprime; Model: Isoprime100; Manufacturer: Isoprime Limited, Cheadle, UK) coupled with an Elemental Analyzer (EA) (Make: Elementar; Model: Vario Isotope Cube; Manufacturer: Isoprime Limited UK) operated using the Ion Vantage Build 1,7,3,0 software. Samples placed in the carousel of EA were combusted at 950 °C in a reactor packed with tungsten (VI) oxide. The IRMS was calibrated using a three-point linear normalization with three certified reference materials: beet sugar IA-R005^13^C (*δ*^13^C value −26.03 ± 0.11‰), protein (casein) (*δ*^13^C value −26.98 ± 0.13‰), and cane sugar IA-R005 ^13^C (*δ*^13^C value −11.64 ± 0.03‰). The *δ*^13^C values of bulk honey and its protein fraction were calculated against this calibration curve and results were expressed in permil (‰). To monitor the stability of the reference gas value and to prevent any drifts from the initial calibration of the IRMS, the protein (casein) standard was run before performing the analysis. The oxide by-products were removed in a reduction reactor containing reduced copper at 650 °C. The carrier gas (helium) flow was 125 mL/min with a total run time of 8 min.

#### 2.3.2. LC-IRMS

An isotopic analysis of the individual sugars fructose (*δ*^13^C_fru_), glucose (*δ*^13^C_glu_), disaccharides (*δ*^13^C_ds_), and trisaccharides (*δ*^13^C_ts_) and foreign oligosaccharides (FOs) of honey was carried out using an IRMS (Isoprime; Isoprime100) coupled with HPLC (Make: Agilent; Model: 1260 infinity; Manufacturer: Agilent Technologies Singapore Pte Ltd., Singapore) as per the FSSAI official method of analysis [28]. IRMS was operated using the Ion Vantage Build 1,7,3,0 software, and HPLC was operated through the Chemstation Open Lab CDS C.01.07 software. The IRMS was calibrated using a three-point linear normalization with three certified reference standards: beet sugar IA-R005^13^C (*δ*^13^C value −26.03‰ ± 0.11‰), glucose (*δ*^13^C value −10.76 ± 0.04‰), and cane sugar IA-R005^13^C (*δ*^13^C value −11.64‰ ± 0.03‰). Internal calibrated standards of fructose, glucose, sucrose, and raffinose with *δ*^13^C values of −11.78 ± 0.4‰, −11.12 ± 0.4‰, −10.90 ± 0.3‰, and −22.20 ± 0.3‰, respectively, were used during this study. The sugars were eluted on a column (Agilent Hi-Plex Ca 300 × 7.7 mm, 8 µm, Manufacturer: Agilent Technologies Singapore Pte Ltd., Singapore) maintained at a temperature of 80 °C. A mobile phase of 100% ultrapure water was used at a flow rate of 0.3 mL/min to separate the individual mono- and disaccharide sugars in honey. The total run time was 45 min and the injection volume was 10 μL. Once the analytes elute from the column, they pass through the LC Liquiface interface (Isoprime) where the oxidation reagent, 20% sodium peroxodisulfate, is mixed with the mobile phase at a flow rate of 12 mL/min to quantitatively oxidize organic material to produce CO_2_ inside the reactor at 95 °C.

### 2.4. Sample Preparation and Analysis

All samples were liquefied at 40 °C, homogenized in an ultrasonic bath for 30 min, and filtered through a nylon mesh material (100–150#) prior to analysis.

#### 2.4.1. *δ*^13^C Value of Honey and Protein by EA-IRMS

Protein extraction: About 10 g of honey samples filtered through nylon mesh material (100–150#) were weighed in a 50 mL centrifuge tube and mixed well with 4 mL ultrapure water. A freshly prepared solution of 10% *w*/*w* sodium tungstate in 0.335 M sulfuric acid was added to each tube and mixed thoroughly. The centrifuge tubes were kept in a water bath at 80 °C until visible protein flocs formed. During the heating process, the tubes were swirled for 20 s at 5–10 min intervals. If no visible floc formed or if the supernatant remained cloudy, then 0.335 M sulfuric acid was added additionally in 2 mL increments with repeated heating after each addition. After the formation of visible flocs, the tubes were centrifuged for 5 min at 6000 rpm and the supernatant was decanted. The precipitate (protein) obtained was rinsed with 40 mL ultrapure water, vortexed, and again centrifuged at 6000 rpm. The centrifugation process was followed by discarding the supernatant, and rinsing was repeated nine times to remove extraneous matter and any residual sugars from the protein precipitate. The protein precipitates were then transferred to a micro-tube and oven dried at 75 °C for 3 h. The dried pellets were crushed with a glass rod to a fine powder and accurately weighed (approximately 0.1–0.2 mg) into tin capsules (dimensions: 4 × 4 × 5 mm) using a micro-analytical balance. Tin capsules were gently folded with the help of forceps avoiding any air trapping and were subjected to EA-IRMS analysis for *δ*^13^C_P_ measurement.

Honey: Similarly, 0.1–0.2 mg honey was weighed into tin capsules (dimensions: 4 × 4 × 5 mm) using a micro-analytical balance. The tin capsules were then gently folded with the help of forceps and were subjected to EA-RMS analysis for *δ*^13^C measurement.

#### 2.4.2. *δ*^13^C Value of Sugars in Honey by LC-IRMS

Twenty milligrams (20 mg) of strained honey sample was weighed into a 15 mL centrifuge tube and dissolved with 5 mL ultrapure water. The solution was sonicated and the volume was made up to 10 mL with ultrapure water. The solution was filtered through 0.22 µm syringe filter into the HPLC injection vial and 10 µL injected into the LC-IRMS.

### 2.5. Stable Carbon Isotopic Value-Based Criteria for Authentic Honey as Defined by the Food Safety and Standards Authority of India (FSSAI) Under FSSR 2018 [25]


**Sr. No.**

**Parameters**

**Criteria**
1Δ*δ*^13^C_P-H_ ‰≥−12C4 Sugar %, max7.03Δ*δ*^13^C_Fru-Glu_ ‰±1.04Δ*δ*^13^C_max_ ‰±2.15Foreign oligosaccharides, percent (%) peak area, max0.7

## 3. Results and Discussion

A total of 98 Indian honey samples were analyzed for the isotopic parameters *δ*^13^C_H_, *δ*^13^C_P_, Δ*δ*^13^C_P-H_, and C4 sugar as per the AOAC method [24] by EA-IRMS, and for *δ*^13^C_Fru-Glu_, Δ*δ*^13^C_max_ (the maximum difference observed between all possible isotopic ratios measured ∆*δ*^13^C_fru-ds_, ∆*δ*^13^C_fru-ts_, ∆*δ*^13^C_fru-P_, ∆*δ*^13^C_glu-ds_, ∆ *δ*^13^C_glu-ts_, ∆*δ*^13^C_glu-P_, ∆*δ*^13^C _ds-ts_, ∆*δ*^13^C_ds-P_, ∆*δ*^13^C_ts-P_), and foreign oligosaccharides (FOs) according to the FSSAI official method of analysis published in 2020 [28], using EA-LC-IRMS at the ISO 17025 [29] accredited laboratory of the Punjab Biotechnology Incubator, SAS Nagar (Mohali), Punjab. The results of all the parameters analyzed by EA-IRMS and LC-IRMS are presented in Supplementary Table S1.

### 3.1. δ^13^C Value for Honey by EA-IRMS

In the present study, the *δ*^13^C_H_ and *δ*^13^C_P_ values for 98 Indian honey samples ranged from −22.07 to −29.02‰ and −22.70 to −27.63‰, respectively. A comparison of the results with the published literature indicates that 95% (*n* = 93) of Indian honey samples fall within the overall published range of −22.7 to −29.49‰ (Table 2). These criteria are widely used to detect honey adulteration in studies and research conducted at the global level [30,31,32,33,34,35,36].

### 3.2. Difference in δ^13^C Values of Protein and Honey (Δδ^13^C_P-H_) and C4 Sugar by EA-IRMS

The *δ*^13^C_P_ and *δ*^13^C_H_ values should be similar for authentic honey, as both honey and protein originate from the same source. Any addition of exogenous sugar will alter the *δ*^13^C value of honey but not its protein fraction, as sugar syrups do not contain any significant amount of protein. This indicates that any adulteration of honey with C4 sugars impacts the *δ*^13^C_H_ value, making it less negative, due to which Δ*δ*^13^C_P-H_ becomes more negative than −1‰ which corresponds to more than 7% C4 sugar addition [18,21,31].

The values of Δ*δ*^13^C_P-H_ and C4 sugar (calculated) for 98 Indian honey samples ranged from −5.20 to 2.50‰ and −19.08 to 28.78%, respectively. The results of these two parameters were compared with the Food Safety and Standards Regulations FSSAI [25], AOAC 998.12 [20], and the authenticity criteria proposed by White et al. in 1998 [18] and Elflein and Raezke in 2008 [19], being used in global trade (i.e., Δ*δ*^13^C_P-H_ ≥ −1‰ and C4 sugar 7% max). As per these criteria, it was found that 94% (*n* = 92) of the samples complied with the criteria for Δ*δ*^13^C_P-H_ and C4 sugar. The traceability of honey samples with the values outside the specified range revealed that they were extracted from the bee colonies fed with exogenous sugar. These findings are in line with the study conducted by Guler et al. in 2014 [44], which reported that when bees are overfed with a glucose-rich diet, they are unable to properly hydrolyze the feeding sugar into honey sugars (fructose and glucose), leading to variations in *δ*^13^C values.

**Figure 2:**
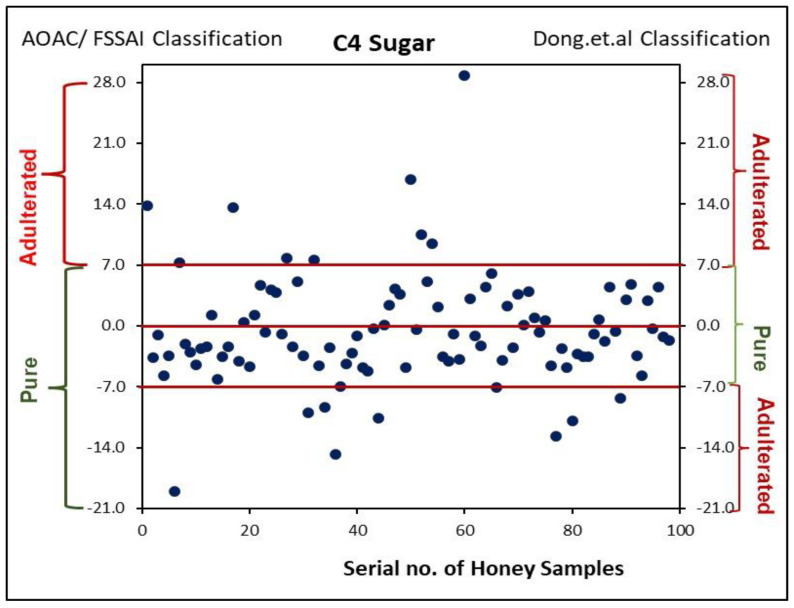
Comparison of results of Indian honey samples with AOAC [20]/FSSAI [25] and Dong et al.’s criteria [41].

As per the AOAC 998.12 method, negative values of C4 sugar are taken as 0% adulteration; whereas, as per Dong et al. [41], samples with C4 sugar beyond ±7% are considered adulterated. On applying this criterion, 82% of the samples were found to be pure against 94% of the samples as per the AOAC criteria (Figure 2).

### 3.3. Δδ^13^C_Fru-Glu_ by LC-IRMS

**Figure 3:**
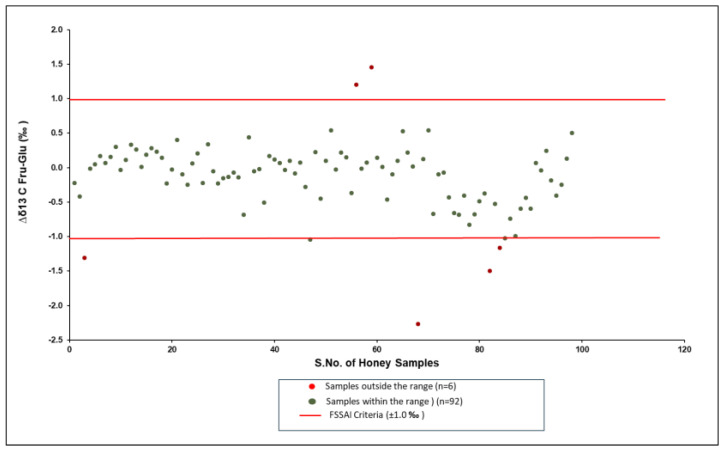
Scatter diagram of Δδ^13^C_Fru-Glu_ values of Indian honey with respect to FSSR 2011 (Amendment 2018).

According to the Food Safety and Standards Regulation of FSSAI [25], Elflein and Raezke [19], and Cabanero et al. [3], the Δδ^13^C_Fru-Glu_ values of authentic honey samples should range from −1.0 to +1.0‰. The Δδ^13^C_Fru-Glu_ of Indian honey ranged from −2.3 to 1.5‰. In this study, 94% (*n* = 92) of the samples were found to comply with said criteria, with values ranging from −1.0 to 0.5‰ (Figure 3). The traceability of samples shows that 6% of the samples not complying with the criteria were extracted from bee colonies fed with exogenous sugar.

### 3.4. Δδ^13^C_max_ by EA/LC-IRMS

**Figure 4:**
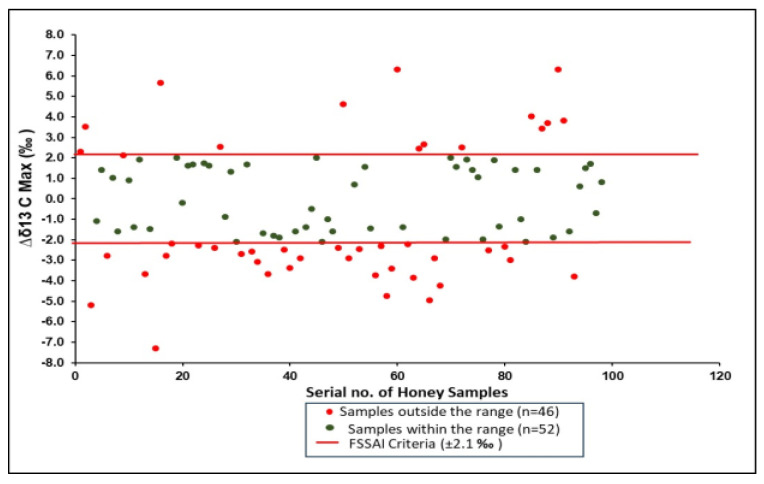
Scatter diagram of Δδ^13^C_max_ values of Indian honey with respect to FSSR 2011 (Amendment 2018).

According to the FSSR [25], and Elflein and Raezke [19], the Δ*δ*^13^C_max_ values of authentic honey samples should vary between −2.1 to +2.1‰. Δ*δ*^13^C_max_ is the maximum difference observed between all possible isotopic ratios measured: ∆*δ*^13^C_Fru-ds_, ∆*δ*^13^C_Fru-ts_, ∆*δ*^13^C_Fru-P_, ∆*δ*^13^C_Glu-ds_, ∆*δ*^13^C_Glu-ts_, ∆*δ*^13^C_Glu-P_, ∆*δ*^13^C_ds-ts_, ∆*δ*^13^C_ds-P_, ∆*δ*^13^C_ts-P._ The *δ*^13^C values of Indian honey samples analyzed for the individual sugars fructose, glucose, disaccharides, and trisaccharides ranged from −21.66 to −29.75‰; from −21.62 to−28.82‰; from −20.32 to −30.99‰; and from−18.30 to −27.12‰, respectively. When Δ*δ*^13^C_max_ was calculated, 47% (*n* = 46) of Indian honey samples fell outside the criteria of ±2.1‰ (Figure 4).In total, 32% of samples are non-compliant due to the larger difference between *δ*^13^C_ds_ and *δ*^13^C_P_. Further, it was observed that Δ*δ*^13^C_max_ values were mainly affected by *δ*^13^C_ds_ values, which were relatively more negative than other individual sugars and also affected the differences in other sugars.

Similar findings are reported by Dong et al., 2017 [30], wherein the *δ*^13^C_ds_ value was more negative than the *δ*^13^C_Glu_ and *δ*^13^C_Fru_ values. A more negative *δ*^13^C_ds_ value than monosaccharide values is most likely due to the specific kinetic isotope effects during biosynthesis [3,30] expected for metabolites further down the carbohydrate metabolic pathway.

The results are also in agreement with the comprehensive study conducted by Xu et al. in 2020 [39] in China, wherein 70% of samples failed to qualify based on the Δ*δ*^13^C_max_ limits due to much higher differences in values between *δ*^13^C_ds_ and *δ*^13^C_P_, although they were 100% natural. The carbon stable isotope ratio values of honey might be affected by many factors, such as climate zone, temperature, rainfall values, and harvesting period. The impact of such factors on the carbon stable isotope ratio values of honey was also reported by Karabagias et al. [9]. Also, a study by White and Winter [21] demonstrated that when bees were artificially fed sugar syrups during the early spring period to build strength, the protein content added by the bees to their stores reflected the isotopic composition of the feed. This isotopic signature could then be detected in the honey if harvested during that time period. The results of the present study for Δδ^13^C_max_ compel further study taking into account bee feeding practices. The results of the current study indicate that Δ*δ*^13^C_max_ needs deeper study to see the effect of various sugar syrups (exogenous or bee feeding) on the Δ*δ*^13^C_max_ value.

### 3.5. Correlation Between δ^13^C Values by EA-IRMS (Δδ^13^C_P-H_) and LC-IRMS (Δδ^13^C_max_, FO)

According to the FSSR [25], Elfelin and Raezkae [19], and AOAC 998.12 criteria [20], honey with a Δ*δ*^13^C_P-H_ greater than or equal to −1‰ and a C4 sugar content of less than 7% is classified as pure. However, in the present study, an interesting observation was made that honey samples with a Δ*δ*^13^C_P-H_ more than +1‰ and a C4 sugar content more negative than 7% also did not meet the Δ*δ*^13^C_max_ and/or foreign oligosaccharide criteria, which could indicate potential adulteration with C3 sugars. These observations suggest that the Δ*δ*^13^C_P-H_ values ‰ (>+1‰equivalent to C4 sugar < −7%) could be an indicator of C3 adulteration to some extent.

### 3.6. Foreign Oligosaccharide by LC-IRMS

**Figure 5:**
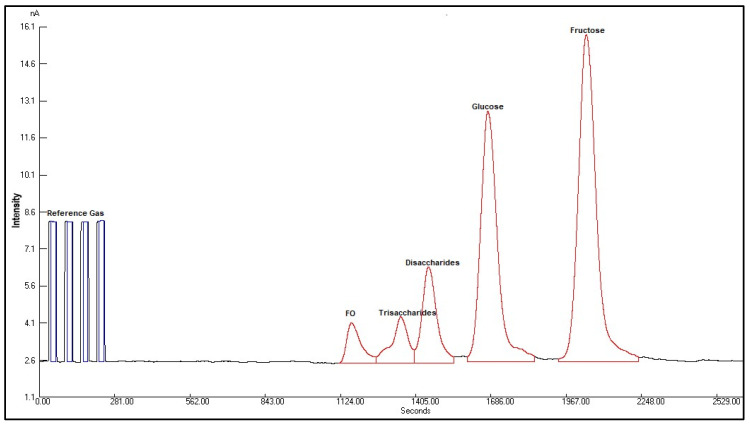
LC-IRMS chromatogram of a sample showing the foreign oligosaccharide peak.

**Figure 6:**
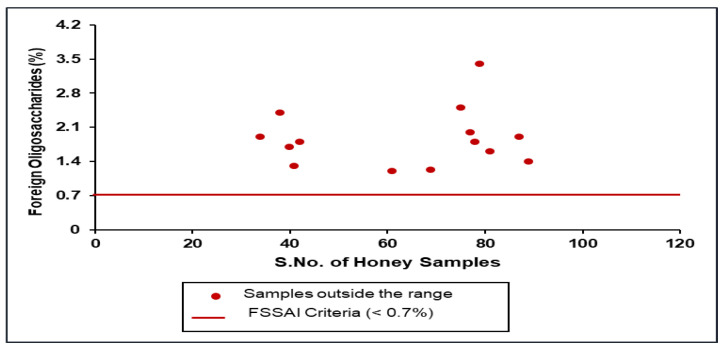
Scatter diagram of foreign oligosaccharides of Indian honey with respect to FSSR 2011 (Amendment 2018).

Elflein and Raezke [19] reported that honeys detected with oligosaccharide peaks could indicate the presence of exogenous C3 sugar syrups, due to the inversion process, and they should not be present in authentic honeys (Figure 5). In the present study, 86% (*n* = 84) of Indian honey samples were found to comply with the foreign oligosaccharide criteria (peak area 0.7% max) as per the FSSAI and Elflein and Raezke [19] (Figure 6).

## 4. Conclusions

The present study conducted on 98 authentic honey samples collected from different geographical and botanical sources in India provides a database of isotopic signatures of Indian honey as per the EA/LC-IRMS-based method. The *δ*^13^C_H_ value of Indian honey ranges from −22.07 to −29.02‰ while the *δ*^13^C_p_ value varies from −22.70 to −27.63‰. It has been observed that 94% (*n* = 92) of Indian honey samples met the Indian regulatory criteria laid down by the Food Safety and Standards Authority of India for Δ*δ*^13^C_P-H_ (≥−1.0‰), Δ*δ*^13^C_Fru-Glu_ (±1‰), and C4 sugar content (7% maximum), with negative C4 sugar values treated as 0% as prescribed by the AOAC 998.12 method. Similarly, 86% (*n* = 86) samples met the criteria of a maximum 0.7% peak area of foreign oligosaccharides (FOs). These observations provide scientific backing for the parameters Δ*δ*^13^C_P-H_, C4 sugar content, Δ*δ*^13^C_Fru-Glu_, and foreign oligosaccharides mentioned in the Food Safety and Standards Regulations of India. However, the non-compliance of a high number (47%) of authentic honey samples for Δ*δ*^13^Cmax (±2.1‰) compels further systematic investigation of the ∆*δ*^13^C_max_ parameter with a special focus on bee feeding practices like the type of feeding material, timing of bee feeding, etc.

Another interesting observation was made in this study. According to the FSSAI and other published literature, honey with a Δ*δ*^13^C_P-H_ greater than or equal to −1‰ and a C4 sugar content of less than 7% is classified as pure. However, in the present study, it was observed that honey samples with a Δ*δ*^13^C_P-H_ of more than +1‰ and a C4 sugar content more negative than −7% also did not meet the Δ*δ*^13^C_max_ and/or foreign oligosaccharide criteria, which could indicate potential adulteration with C3 sugars. These observations suggest that the Δδ^13^C_P-H_ values (>+1‰ equivalent to C4 sugar < −7%) could be an indicator of C3 adulteration to some extent.

## Figures and Tables

**Table 1:** Botanical origin-wise details of honey samples.

Sr. No.	Type of Flora	Number of Samples
A	Multifloral	41
B	Monofloral	57
1	Mustard	10
2	Eucalyptus Tree	7
3	Rosewood (Shisham) Tree	7
4	Lychee Tree	6
5	Rubber Tree	6
6	Coriander	4
7	Carom	3
8	Acacia Tree	2
9	Clover (Trifolium)	2
10	Drum Stick Tree (Moringa)	2
11	Indian Jujube	1
12	Bitter Vine	1
13	Plectranthus (Chhichri)	1
14	Coconut	1
15	Gum Arabic Tree (Babool)	1
16	Herbs	1
17	Sesame	1
18	Sunflower	1
Total		98

**Table 2:** Summary of *δ*^13^C_H_ from different countries as per the literature viz-a-viz Indian honey.

Sr. No.	Name of Countries	*δ*^13^C_H_ Value (‰)	Previous Studies
1	Thailand (*n* = 49)	−23.10 to −29.10	[37]
2	Lebanon (*n* = 33)	−24.50 to −26.50	[31]
3	Romanian (*n* = 48)	−24.47 to −27.0	[38]
4	China (*n* = 94)	−23.21 to −29.49	[39]
5	Turkey (*n* = 19)	−25.20 to −26.10	[40]
6	China (*n* = 53)	−23.55 to −27.44	[41]
7	China (*n* = 800)	−24.20 to −25.85	[30]
8	Turkey (*n* = 100)	−22.70 to −27.4	[42]
9	Turkey (*n* = 31)	−23.30 to −27.58	[43]
10	Argentina, Austria, Bulgaria, Canada, China, Czech Republic, El Salvador, France, Germany, Great Britain, Greece, Hungary, India, Italy, Japan, Malaysia, Mexico, Romania, Slovakia, Slovenia, Spain, Turkey, Ukraine, Uruguay, Vietnam (*n* = 451)	−23.0 to −27.3	[19]
11	United States (ASCS), Germany, UK, Mexico, Italy, Spain (*n* = 224)	>−23.5	[18]
Overall published range in the literature		−22.70 to −29.49	--
Overall observed range of Indian honey (*n* = 98) in this study		−22.07 to −29.02	--
Observed range of Indian honey in this study falling under published ranges in the literature (*n* = 93)		−23.24 to −29.02	--
Observed range of Indian honey in this study not falling under published ranges in the literature (*n* = 5)		−22.07 to −22.56	

## Data Availability

The original contributions presented in the study are included in the article/Supplementary Material, further inquiries can be directed to the corresponding author.

## Supplementary Materials

The following supporting information can be downloaded at https://www.mdpi.com/article/10.3390/foods14081289/s1, Table S1: EA/LC-IRMS results of 98 samples of Indian honey.

## References

[1] Terrab A., González A.G., Díez M.J., Heredia F.J. (2003). Characterisation of Moroccan unifloral honeys using multivariate analysis. Eur. Food Res. Technol..

[2] Chen L., Xue X., Ye Z., Zhou J., Chen F., Zhao J. (2011). Determination of Chinese honey adulterated with high fructose corn syrup by near infrared spectroscopy. Food Chem..

[3] Cabanero A.I., Recio J.L., Ruperez M. (2006). Liquid chromatography coupled to isotope ratio mass spectrometry: A new perspective on honey adulteration detection. J. Agric. Food Chem..

[4] Padovan G.J., De Jong D., Rodrigues L.P., Marchini J.S. (2003). Detection of adulteration of commercial honey samples by the 13C/12C isotopic ratio. Food Chem..

[5] Padovan G.J., Rodrigues L.P., Leme I.A., De Jong D., Marchini J.S. (2007). Presence of C4 Sugars in Honey Samples Detected by the Carbon Isotope Ratio Measured by IRMS. Eurasian J. Anal. Chem..

[6] Ruiz-Matute A.I., Weiss M., Sammataro D., Finely J., Sanz M.L. (2010). Carbohydrate composition of high-fructose corn syrups (HFCS) used for bee feeding: Effect on honey composition. J. Agric. Food Chem..

[7] Földhádzi G. (1994). Analysis and Quantitation of Sugars in Honey of Different Botanical Origin Using High Performance Liquid Chromatography. https://www.cabidigitallibrary.org/doi/full/10.5555/19970200094.

[8] Ruiz-Matute A.I., Soria A.C., Martínez-Castro I., Sanz M.L. (2007). A new methodology based on GC−MS to detect honey adulteration with commercial syrups. J. Agric. Food Chem..

[9] Karabagias I.K., Casiello G., Kontakos S., Louppis A.P., Longobardi F., Kontominas M.G. (2016). Investigating the impact of botanical origin and harvesting period on carbon stable isotope ratio values (13C/12C) and different parameter analysis of Greek unifloral honeys: A chemometric approach for correct botanical discrimination. Int. J. Food Sci. Technol..

[10] Cordella C., Militão J.S., Clément M.C., Drajnudel P., Cabrol-Bass D. (2005). Detection and quantification of honey adulteration via direct incorporation of sugar syrups or bee-feeding: Preliminary study using high-performance anion exchange chromatography with pulsed amperometric detection (HPAEC-PAD) and chemometrics. Anal. Chim. Acta.

[11] Herpai Z., Szigeti J., Csapó J. (2013). A rapid and sensitive method for the determination of high-fructose corn syrup (HFCS) in honey. Acta Univ. Sapientiae Aliment..

[12] Wang S., Guo Q., Wang L., Lin L., Shi H., Cao H., Cao B. (2015). Detection of honey adulteration with starch syrup by high performance liquid chromatography. Food Chem..

[13] Li S., Zhang X., Shan Y., Su D., Ma Q., Wen R., Li J. (2017). Qualitative and quantitative detection of honey adulterated with high-fructose corn syrup and maltose syrup by using near-infrared spectroscopy. Food Chem..

[14] Ždiniaková T., Loerchner C., De R.O., Dimitrova T., Kaklamanos G., Breidbach A., Maquet A. (2023). EU Coordinated Action to Deter Certain Fraudulent Practices in the Honey Sector. JRC Publications Repository. https://joint-research-centre.ec.europa.eu.

[15] White J.W., Doner L.W. (1978). Mass spectrometric detection of high-fructose corn sirup in honey by use of 13C/12C ratio: Collaborative study. J. Assoc. Off. Anal. Chem..

[16] White J.W., Doner L.W. (1978). The 13C/12C ratio in honey. J. Apic. Res..

[17] Winkler F.J., Schmidt H.L. (1980). Scope of the application of 13C isotope mass spectrometry in Food Analysis. Z. Fuer Lebensm.-Unters. Und-Forsch..

[18] White J.W., Winters K., Peter M., Rossmann A. (1998). Stable carbon isotope ratio analysis of honey: Validation of internal standard procedure for worldwide application. J. AOAC Int..

[19] Elflein L., Raezke K.P. (2008). Improved detection of honey adulteration by measuring differences between 13C/12C stable carbon isotope ratios of protein and sugar compounds with a combination of elemental analyzer-isotope ratio mass spectrometry and liquid chromatography-isotope ratio mass spectrometry δ13C-EA/LC-IRMS). Apidologie.

[20] Cunniff P., AOAC Official (1999). Method 998.12. C-4 plant sugars in honey. Official Methods of Analysis of AOAC International.

[21] White J.W., Winters K. (1989). Honey protein as internal standard for stable carbon isotope ratio detection of adulteration of honey. J. Assoc. Off. Anal. Chem..

[22] White J.W. (1992). Internal standard stable carbon isotope ratio method for determination of C-4 plant sugars in honey: Collaborative study, and evaluation of improved protein preparation procedure. J. AOAC Int..

[23] Food and Agriculture Organization of the United Nations (2012). Turkish Food Codex-Directive on Honey.

[24] AGMARK Official Gazatte (2024). Honey Grading and Marking Rules.

[25] Food Safety and Standards Authority of India (2023). Food Safety and Standards Authority of India. Food Products Standards and Food Additives, Sweetening Agents Including Honey. Food Safety and Standards Regulation 2011 (Amended in 2018).

[26] True Source Certified^®^ Standards V7.3.1, Revision Date: 1 March 2024. www.truesourcehoney.com.

[27] (2024). Food Authenticity-Determination of the δ^13^C Value of Mono-(Fructose and Glucose), di-, and trisaccharides in Honey by Liquid Chromatography-Isotope Ratio Mass Spectrometry (LC-IRMS).

[28] FSSAI Official Method of Analysis (2020). Method for the Estimation of ∆δ13Cfru-glu, ∆δ13Cmax and Foreign Oligosaccharides in Honey by Elemental Analysis (EA)/Liquid Chromatography (LC)-Isotopic Ratio Mass Spectrometry (EA/LC-IRMS).

[29] (2017). General Requirements for the Competence of Testing and Calibration Laboratories.

[30] Dong H., Xiao K., Xian Y. (2017). Isotope ratio mass spectrometry coupled to element analyzer and liquid chromatography to identify commercial honeys of various botanical types. Food Anal. Methods.

[31] El Hawari K., Al Iskandarani M., Jaber F., Ezzeddine R., Ziller L., Perini M., Bontempo L., Pellegrini M., Camin F. (2021). Evaluation of honey authenticity in Lebanon by analysis of carbon stable isotope ratio using elemental analyzer and liquid chromatography coupled to isotope ratio mass spectrometry. J. Mass Spectrom..

[32] Geană E.I., Ciucure C.T., Costinel D., Ionete R.E. (2020). Evaluation of honey in terms of quality and authenticity based on the general physicochemical pattern, major sugar composition and δ13C signature. Food Control.

[33] Kelly J.D., Petisco C., Downey G. (2006). Application of Fourier transform midinfrared spectroscopy to the discrimination between Irish artisanal honey and such honey adulterated with various sugar syrups. J. Agric. Food Chem..

[34] Khatun M.A., Yoshimura J., Yoshida M., Suzuki Y., Huque R., Kelly S.D., Munshi M.K. (2024). Isotopic characteristics (δ13C, δ15N, and δ18O) of honey from Bangladesh retail markets: Investigating sugar manipulation, botanical and geographical authentication. Food Chem..

[35] Rogers K.M., Sim M., Stewart S., Phillips A., Cooper J., Douance C., Pyne R., Rogers P. (2014). Investigating C-4 sugar contamination of manuka honey and other New Zealand honey varieties using carbon isotopes. J. Agric. Food Chem..

[36] Zhou X., Taylor M.P., Salouros H., Prasad S. (2018). Authenticity and geographic origin of global honeys determined using carbon isotope ratios and trace elements. Sci. Rep..

[37] Kamdee K., Naksuriyawong S., Uapoonphol N., Fungklin R., Esor J., Permnamtip V., Meepho S.-A., Judprasong K. (2023). Evaluation of honey authenticity in Thailand by analysis of carbon stable isotope ratio using elemental analyser coupled to isotope ratio mass spectrometry and cavity ring-down spectrometry. Int. J. Food Sci. Technol..

[38] Geana E.I., Ciucure C.T. (2020). Establishing authenticity of honey via comprehensive Romanian honey analysis. Food Chem..

[39] Xu J., Liu X., Wu B., Cao Y. (2020). A comprehensive analysis of 13C isotope ratios data of authentic honey types produced in China using the EA-IRMS and LC-IRMS. J. Food Sci. Technol..

[40] Cengiz M.M., Tosun M., Topal M. (2018). Determination of the physicochemical properties and 13C/12C isotope ratios of some honeys from the northeast Anatolia region of Turkey. J. Food Compos. Anal..

[41] Dong H., Luo D., Xian Y., Luo H., Guo X., Li C., Zhao M. (2016). Adulteration identification of commercial honey with the C-4 sugar content of negative values by an elemental analyzer and liquid chromatography coupled to isotope ratio mass spectroscopy. J. Agric. Food Chem..

[42] Çinar S.B., Ekşi A., Coşkun İ. (2014). Carbon isotope ratio (13C/12C) of pine honey and detection of HFCS adulteration. Food Chem..

[43] Simsek A., Bilsel M., Goren A.C. (2012). 13C/12C pattern of honey from Turkey and determination of adulteration in commercially available honey samples using EA-IRMS. Food Chem..

[44] Guler A., Kocaokutgen H., Garipoglu A.V., Onder H., Ekinci D., Biyik S. (2014). Detection of adulterated honey produced by honeybee (*Apis mellifera* L.) colonies fed with different levels of commercial industrial sugar (C3 and C4 plants) syrups by the carbon isotope ratio analysis. Food Chem..

