# Supplementary material for: Authentication of Indian Honey Based on Carbon Stable Isotope Ratio Analysis—Verification of Indian Regulatory Criteria
Source: Foods. 2025 Apr 8;14(8):1289. doi: 10.3390/foods14081289 (PMC12026366; doi:10.3390/foods14081289)
Supplement: Supplementary file 1 [file foods-14-01289-s001.zip › foods-3466611-supplementary.pdf]

**Authentication of Indian Honey based on Carbon Stable Isotope Ratio Analysis—Verification of Indian Regulatory Criteria**

## Supplementary Information

**Table S1: EA/LC-IRMS results of 98 samples of Indian honey.**

| Sample Code | Location | Source | $\delta^{13} C_P$ | $\delta^{13} C_H$ | $\Delta\delta^{13} C_{P-H}$ | $C_4$ Sugar | $\Delta\delta^{13} C_{Fru-Glu}$ | $\Delta\delta^{13}C_{max}$ | FO |
|---|---|---|---|---|---|---|---|---|---|
| | | | | **Mustard Honey (n=10)** | | | | | |
| H01 | Haryana | Beekeeper | -26.67 | -26.60 | -0.1 | 0.4 | -0.2 | 2.0 | ND |
| H02 | Maharashtra | CBRTI* | -25.89 | -25.70 | -0.2 | 1.2 | 0.4 | 1.6 | ND |
| H03 | Haryana | AICRP** | -25.83 | -25.21 | -0.6 | 3.8 | 0.2 | 1.6 | ND |
| H04 | Rajasthan | AICRP | -26.22 | -24.94 | -1.3 | 7.7 | 0.3 | 2.5 | ND |
| H05 | Haryana | Beekeeper | -26.78 | -25.91 | -0.9 | 5.1 | 0.2 | -2.5 | ND |
| H06 | Rajasthan | Beekeeper | -25.81 | -24.29 | -1.5 | 9.4 | 0.1 | 1.5 | ND |
| H07 | Rajasthan | Beekeeper | -26.99 | -26.61 | -0.4 | 2.2 | -0.4 | -1.5 | ND |
| H08 | Punjab | Beekeeper | -26.23 | -26.39 | 0.2 | -1.0 | 0.1 | -4.8 | ND |
| H09 | Rajasthan | Beekeeper | -25.59 | -26.72 | 1.1 | -7.1 | 0.2 | -5.0 | ND |
| H10 | Haryana | Beekeeper | -26.54 | -26.62 | 0.1 | -0.5 | 0.5 | -2.9 | ND |
| | **Mean** | | -26.26 | -25.90 | -0.27 | 2.12 | 0.14 | -0.75 | ND |
| | **SD** | | 0.47 | 0.85 | 0.69 | 4.73 | 0.27 | 2.92 | ND |
| | **Range** | | -25.99to -26.99 | -24.29 to -26.72 | -1.5 to 1.1 | -7.1 to 9.4 | -0.4 to 0.4 | -5.0 to2.5 | ND |
| | | | | **Eucalyptus Honey (n=7)** | | | | | |
| H11 | Punjab | Beekeeper | -27.30 | -27.72 | 0.4 | -2.4 | 0.3 | 5.6 | ND |
| H12 | Punjab | Beekeeper | -25.25 | -25.85 | 0.60 | -3.9 | 1.5 | -3.4 | ND |
| H13 | Punjab | Beekeeper | -27.63 | -22.47 | -5.2 | 28.8 | 0.1 | 6.3 | ND |
| H14 | Uttar Pradesh | Beekeeper | -25.20 | -25.82 | 0.6 | -4.0 | 0.0 | -2.9 | ND |
| H15 | Odisha | Beekeeper | -27.04 | -26.26 | -0.8 | 4.5 | -0.3 | 1.7 | ND |

| | | | | | | | | | |
|---|---|---|---|---|---|---|---|---|---|
| H16 | Odisha | Beekeeper | -25.62 | -25.82 | 0.2 | -1.3 | 0.1 | -0.7 | ND |
| H17 | Odisha | Beekeeper | -24.67 | -24.93 | 0.3 | -1.7 | 0.5 | 0.8 | ND |
| | **Mean** | | -26.10 | -25.55 | -0.56 | 2.86 | 0.31 | 1.06 | ND |
| | **SD** | | 1.19 | 1.60 | 2.10 | 11.79 | 0.58 | 3.81 | ND |
| | **Range** | | - 24.67 to - 27.63 | -22.47 to - 27.72 | -5.2 to 0.6 | -4.0 to 28. 8 | -0.3 to 1.5 | -3.4 to 6.3 | ND |
| **Rosewood (Shisham) Honey (n=7)** | | | | | | | | | |
| H18 | Maharashtra | CBRTI | -26.54 | -25.76 | -0.8 | 4.6 | -0.1 | 1.7 | ND |
| H19 | Punjab | Beekeeper | -25.23 | -25.78 | 0.6 | -3.5 | 1.2 | -3.8 | ND |
| H20 | Punjab | Beekeeper | -25.24 | -25.88 | 0.6 | -4.1 | 0.0 | -2.3 | ND |
| H21 | Punjab | AICRP | -24.75 | -25.15 | 0.4 | -2.7 | 0.1 | -1.4 | ND |
| H22 | Rajasthan | AICRP | -24.88 | -25.25 | 0.4 | -2.4 | 0.3 | 1.9 | ND |
| H23 | Rajasthan | AICRP | -25.56 | -25.67 | 0.1 | -0.7 | -0.2 | -2.3 | ND |
| H24 | Rajasthan | Beekeeper | -25.73 | -23.52 | -2.2 | 13.8 | -0.2 | 2.3 | ND |
| | **Mean** | | -25.42 | -25.29 | -0.13 | 0.71 | 0.16 | -0.56 | ND |
| | **SD** | | 0.60 | 0.83 | 1.03 | 6.46 | 0.49 | 2.47 | ND |
| | **Range** | | -24.75 to - 26.54 | -23.52 to - 25.88 | -2.2 to 0.6 | -4.1 to 13.8 | -0.2 to1.2 | -3.8 to 2.3 | ND |
| **Lychee Honey (n=6)** | | | | | | | | | |
| H25 | UP | Beekeeper | -24.49 | -22.48 | -2.0 | 13.6 | 0.2 | -2.8 | ND |
| H26 | Himachal Pradesh | Beekeeper | -25.30 | -24.69 | -0.6 | 3.9 | -0.1 | 2.5 | ND |
| H27 | UP | Beekeeper | -24.65 | -24.51 | -0.1 | 0.9 | -0.1 | 1.9 | ND |
| H28 | Assam | Beekeeper | -25.50 | -25.62 | 0.1 | -0.8 | -0.4 | 1.4 | ND |
| H29 | Assam | Beekeeper | -25.46 | -25.36 | -0.1 | 0.6 | -0.7 | 1.1 | 2.5 |
| H30 | Assam | AICRP | -26.82 | -27.61 | 0.8 | -4.6 | -0.7 | -2.0 | ND |
| | **Mean** | | -25.37 | -25.05 | -0.32 | 2.27 | -0.30 | 0.35 | 1.80 |

| | | | | | | | | | |
|---|---|---|---|---|---|---|---|---|---|
| | **SD** | | 0.83 | 1.67 | 0.94 | 6.20 | 0.36 | 2.20 | ND |
| | **Range** | | -24.49 to - 26.82 | -22.48 to - 27.61 | -2.0 to 0.8 | 3.9 to 13.6 | -0.7 to 0.2 | -2.8 to 1.4 | |
| **Rubber Honey (n=6)** | | | | | | | | | |
| H31 | Kerala | AICRP | -23.54 | -23.97 | 0.4 | -3.1 | 0.2 | -2.5 | ND |
| H32 | Kerala | AICRP | -23.65 | -23.64 | 0.0 | 0.1 | 0.1 | 2.0 | ND |
| H33 | Tamil Nadu | Beekeeper | -26.02 | -28.09 | 2.1 | -12.7 | -0.4 | -2.5 | 2.0 |
| H34 | Kerela | Beekeeper | -25.21 | -25.62 | 0.4 | -2.6 | -0.8 | 1.9 | 1.8 |
| H35 | Assam | Beekeeper | -25.25 | -25.99 | 0.7 | -4.8 | -0.7 | -1.4 | 3.4 |
| H36 | Assam | Beekeeper | -27.11 | -29.02 | 1.9 | -11.0 | -0.5 | -2.3 | ND |
| | **Mean** | | -25.13 | -26.06 | 0.92 | -5.68 | -0.35 | -0.80 | 2.40 |
| | **SD** | | 1.37 | 2.16 | 0.87 | 5.06 | 0.41 | 2.17 | 0.87 |
| | **Range** | | -23.54to-27.11 | -23.64to-29.02 | 0.0 to2.1 | -12.7to 0.1 | -0.8 to0.2 | -2.5 to2.0 | 1.8 to 3.4 |
| **Coriander Honey (n=4)** | | | | | | | | | |
| H37 | Punjab | AICRP | -25.77 | -26.76 | 1.0 | -6.2 | 0.0 | -1.5 | ND |
| H38 | Madhya Pradesh | Beekeeper | -24.80 | -24.12 | -0.7 | 4.5 | 0.1 | 2.4 | ND |
| H39 | Rajasthan | Beekeeper | -26.12 | -25.53 | -0.6 | 3.6 | 0.5 | 2.0 | ND |
| H40 | Assam | Beekeeper | -24.81 | -24.79 | 0.0 | 0.1 | -0.7 | 1.6 | ND |
| | **Mean** | | -25.38 | -25.30 | -0.08 | 0.50 | -0.03 | 1.13 | ND |
| | **SD** | | 0.67 | 1.13 | 0.78 | 4.85 | 0.50 | 1.78 | ND |
| | **Range** | | -24.80 to-26.12 | -24.12 to-26.76 | -0.7 to1.0 | -6.2to 4.5 | -0.7to0.5 | -1.5 to2.4 | |
| **Carom (Ajwain) Honey (n=3)** | | | | | | | | | |
| H41 | Rajasthan | Beekeeper | -24.20 | -23.75 | -0.4 | 3.1 | 0.0 | -1.4 | 1.2 |
| H42 | Rajasthan | Beekeeper | -25.51 | -25.15 | -0.4 | 2.3 | -2.3 | -4.2 | ND |
| H43 | Maharashtra | Beekeeper | -25.98 | -26.39 | 0.4 | -2.5 | 0.1 | -2.0 | 1.2 |

| | | | | | | | | | |
|---|---|---|---|---|---|---|---|---|---|
| | **Mean** | | -25.23 | -25.10 | -0.13 | 0.97 | -0.73 | -2.53 | 1.20 |
| | **SD** | | 0.92 | 1.32 | 0.46 | 3.03 | 1.36 | 1.47 | 0.00 |
| | **Range** | | -24.20 to-25.98 | -23.75 to-26.39 | -0.4 to0.4 | -2.5to 3.1 | -2.3 to0.1 | -4.2 to-1.4 | |
| **Acacia Honey (n=2)** | | | | | | | | | |
| H44 | Jammu and Kashmir | AICRP | -26.09 | -26.44 | 0.4 | -2.1 | 0.2 | -1.6 | ND |
| H45 | Rajasthan | AICRP | -25.33 | -25.14 | -0.2 | 1.2 | 0.3 | -3.7 | ND |
| | | Mean | -25.71 | -25.79 | 0.10 | -0.45 | 0.25 | -2.65 | ND |
| | | SD | 0.54 | 0.92 | 0.42 | 2.33 | 0.07 | 1.48 | ND |
| **Clover (Trifolium) Honey (n=2)** | | | | | | | | | |
| H46 | Punjab | Beekeeper | -26.63 | -27.59 | 1.0 | -5.7 | 0.0 | -1.1 | ND |
| H47 | Punjab | AICRP | -26.32 | -27.07 | 0.8 | -4.5 | 0.0 | 0.9 | ND |
| | | Mean | -26.48 | -27.33 | 0.90 | -5.10 | 0.0 | -0.10 | ND |
| | | SD | 0.22 | 0.37 | 0.14 | 0.85 | 0.00 | 1.41 | ND |
| **Drumstick (Moringa) Honey (n=2)** | | | | | | | | | |
| H48 | Jammu and Kashmir | AICRP | -24.69 | -25.20 | 0.5 | -3.4 | -0.2 | -2.1 | ND |
| H49 | Tamil Nadu | Beekeeper | -24.54 | -25.38 | 0.8 | -5.7 | 0.2 | -3.8 | ND |
| | | Mean | -24.62 | -25.29 | 0.62 | -4.55 | 0.00 | -2.95 | ND |
| | | SD | 0.11 | 0.13 | 0.21 | 1.63 | 0.28 | 1.20 | ND |
| **Indian Jujube Honey (n=1)** | | | | | | | | | |
| H50 | Rajasthan | AICRP | -22.80 | -25.30 | 2.5 | -19.1 | 0.2 | -2.8 | ND |
| **Bitter vine Honey (n=1)** | | | | | | | | | |
| H51 | Odisha | Beekeeper | -26.53 | -26.05 | -0.5 | 2.9 | -0.2 | 0.6 | ND |
| **Plectranthus (Chhichri) Honey (n=1)** | | | | | | | | | |
| H52 | Himachal Pradesh | Beekeeper | -26.15 | -26.74 | 0.6 | -3.6 | -0.5 | -1.0 | ND |

| | | | | | | | | | |
|---|---|---|---|---|---|---|---|---|---|
| **Coconut Honey (n=1)** | | | | | | | | | |
| H53 | Kerala | AICRP | -23.04 | -25.01 | 1.97 | -14.8 | -0.1 | -3.7 | ND |
| **Gum Arabic (Babool) Honey (n=1)** | | | | | | | | | |
| H54 | Rajasthan | Beekeeper | -25.29 | -24.35 | -0.9 | 6.0 | 0.5 | 2.7 | ND |
| **Herbs Honey (n= 1)** | | | | | | | | | |
| H55 | Punjab | Beekeeper | -26.25 | -26.43 | 0.2 | -1.1 | -1.3 | -5.2 | ND |
| **Sesame Honey (n=1)** | | | | | | | | | |
| H56 | Gujarat | Beekeeper | -26.13 | -26.67 | 0.5 | -3.3 | -0.4 | -3.0 | 1.6 |
| **Sunflower Honey (n=1)** | | | | | | | | | |
| H57 | Punjab | Beekeeper | -24.93 | -22.37 | -2.6 | 16.8 | 0.1 | 4.6 | ND |
| **Multiflora Honey (n=41)** | | | | | | | | | |
| H58 | Punjab | Beekeeper | -25.75 | -26.34 | 0.6 | -3.7 | -0.4 | 3.5 | ND |
| H59 | Jammu and Kashmir | AICRP | -25.18 | -25.72 | 0.5 | -3.5 | 0.1 | 1.4 | ND |
| H60 | Punjab | AICRP | -25.58 | -24.43 | -0.8 | 7.2 | 0.1 | 1.0 | ND |
| H61 | Punjab | AICRP | -25.64 | -26.13 | 0.49 | -3.1 | 0.3 | 2.1 | ND |
| H62 | Punjab | AICRP | -25.27 | -25.83 | 0.6 | -3.6 | 0.2 | -7.3 | ND |
| H63 | Punjab | AICRP | -25.75 | -26.40 | 0.6 | -4.0 | 0.1 | -2.2 | ND |
| H64 | Maharashtra | CBRTI | -25.75 | -26.49 | 0.7 | -4.6 | 0.0 | -0.2 | ND |
| H65 | Punjab | AICRP | -25.08 | -24.44 | -0.6 | 4.2 | 0.1 | 1.7 | ND |
| H66 | Punjab | AICRP | -25.30 | -25.45 | 0.1 | -1.0 | -0.2 | -2.4 | ND |
| H67 | Jammu and Kashmir | AICRP | -25.38 | -25.75 | 0.4 | -2.4 | -0.1 | -0.9 | ND |
| H68 | Jammu and Kashmir | AICRP | -25.79 | -24.98 | -0.8 | 5.0 | -0.2 | 1.3 | ND |
| H69 | Jammu and Kashmir | AICRP | -23.48 | -24.86 | 1.4 | -10.0 | -0.1 | -2.7 | ND |

| | | | | | | | | | |
|---|---|---|---|---|---|---|---|---|---|
| H70 | Jammu and Kashmir | AICRP | -23.62 | -22.56 | -0.4 | 7.6 | -0.1 | 1.7 | ND |
| H71 | Jammu and Kashmir | AICRP | -24.47 | -25.14 | 0.7 | -4.5 | -0.1 | -2.6 | ND |
| H72 | Nagaland | AICRP | -26.06 | -27.60 | 1.5 | -9.4 | -0.7 | -3.1 | ND |
| H73 | Nagaland | AICRP | -26.45 | -26.87 | 0.4 | -2.5 | 0.4 | -1.7 | ND |
| H74 | Kerala | AICRP | -23.18 | -24.12 | 0.9 | -7.0 | 0.0 | -1.8 | ND |
| H75 | Kerala | AICRP | -24.11 | -24.74 | 0.63 | -4.4 | -0.5 | -1.9 | 2.4 |
| H76 | Kerala | AICRP | -23.45 | -23.61 | 0.2 | -1.2 | 0.1 | -3.4 | 1.7 |
| H77 | Kerala | AICRP | -23.35 | -24.00 | 0.6 | -4.8 | 0.1 | -1.6 | 1.3 |
| H78 | Kerala | AICRP | -23.94 | -24.68 | 0.7 | -5.2 | 0.0 | -2.9 | 1.8 |
| H79 | Kerala | AICRP | -23.37 | -23.41 | 0.0 | -0.3 | 0.1 | -1.4 | ND |
| H80 | Kerala | AICRP | -23.04 | -24.45 | 1.41 | -10.6 | -0.1 | -0.5 | ND |
| H81 | Himachal Pradesh | AICRP | -26.91 | -26.50 | -0.41 | 2.4 | -0.3 | -2.1 | ND |
| H82 | Himachal Pradesh | AICRP | -26.27 | -25.57 | -0.70 | 4.2 | -1.0 | -1.0 | ND |
| H83 | Himachal Pradesh | AICRP | -24.74 | -24.20 | -0.5 | 3.6 | 0.2 | -1.6 | ND |
| H84 | Himachal Pradesh | AICRP | -24.74 | -25.46 | 0.7 | -4.8 | -0.4 | -2.4 | ND |
| H85 | Punjab | Beekeeper | -24.82 | -23.24 | -1.58 | 10.4 | 0.0 | 0.7 | ND |
| H86 | Chandigarh | Beekeeper | -25.06 | -25.24 | 0.2 | -1.2 | -0.5 | -2.2 | ND |
| H87 | Jammu and Kashmir | AICRP | -25.30 | -25.65 | 0.4 | -2.3 | -0.1 | -3.9 | ND |
| H88 | Gujarat | Beekeeper | -26.05 | -26.64 | 0.58 | -3.6 | -1.5 | 1.4 | ND |
| H89 | Himachal Pradesh | AICRP | -24.55 | -24.69 | 0.1 | -0.9 | -1.2 | -2.1 | ND |
| H90 | Himachal Pradesh | AICRP | -23.87 | -23.76 | -0.1 | 0.8 | -1.0 | 4.0 | ND |
| H91 | Himachal Pradesh | AICRP | -26.25 | -26.55 | 0.3 | -1.8 | -0.7 | 1.4 | ND |
| H92 | Himachal Pradesh | Beekeeper | -25.97 | -25.25 | -0.7 | 4.4 | -1.0 | 3.4 | 1.9 |

| | | | | | | | | | |
|---|---|---|---|---|---|---|---|---|---|
| H93 | Himachal Pradesh | Beekeeper | -25.75 | -25.85 | 0.1 | -0.6 | -0.6 | 3.7 | ND |
| H94 | Himachal Pradesh | Beekeeper | -24.41 | -25.64 | 1.2 | -8.3 | -0.4 | -1.9 | 1.4 |
| H95 | Himachal Pradesh | AICRP | -24.56 | -24.11 | -0.5 | 3.0 | -0.6 | 6.3 | ND |
| H96 | Andhra Pradesh | AICRP | -22.70 | -22.07 | -0.6 | 4.8 | 0.1 | 3.8 | ND |
| H97 | Tamil Nadu | AICRP | -24.19 | -24.68 | 0.5 | -3.4 | 0.0 | -1.6 | ND |
| H98 | Odisha | Beekeeper | -26.51 | -26.56 | 0.1 | -0.3 | -0.4 | 1.5 | ND |
| | | **Mean** | -24.92 | -25.11 | 0.22 | -1.35 | -0.25 | -0.40 | 1.75 |
| | | **SD** | 1.09 | 1.22 | 0.68 | 4.85 | 0.44 | 2.69 | 0.39 |
| | **Range** | | -22.70 to -26.91 | -22.07 to -26.64 | -1.6 to 1.4 | -10.6 to 10.5 | -1.5 to 0.2 | -3.9 to 6.30 | 1.3 to 2.4 |

* Central Bee Research Training Institute.

** All-India Coordinated Research Project on Honey Bees and Pollinators.

**The parameter-wise-method standard deviation (σ)**

$\delta^{13}$ C $_{P}$: 0.19 ‰;

$\delta^{13}$ C $_{H}$: 0.20 ‰;

$\delta^{13}$ $C_{Fru}$: 0.35‰;

$\delta^{13}$ $C_{Glu}$: 0.45 ‰;

$\delta^{13}$ $C_{ts}$: 0.40 ‰;

$\delta^{13}$ $C_{ds}$: 0.39 ‰.
